# Inferring evolutionary responses of *Anolis carolinensis* introduced into the Ogasawara archipelago using whole genome sequence data

**DOI:** 10.1038/s41598-017-17852-7

**Published:** 2017-12-21

**Authors:** Satoshi Tamate, Watal M. Iwasaki, Kenneth L. Krysko, Brian J. Camposano, Hideaki Mori, Ryo Funayama, Keiko Nakayama, Takashi Makino, Masakado Kawata

**Affiliations:** 1 Department of Ecology and Evolutionary Biology, Graduate School of Life Sciences, Tohoku University, Aoba-ku, Sendai 980-8578 Japan; 2 Department of Evolutionary Studies of Biosystems, SOKENDAI (The Graduate University for Advanced Studies), Hayama, Japan; 3 Division of Herpetology, Florida Museum of Natural History, 1659 Museum Road, University of Florida, Gainesville, FL 32611 USA; 4 Forest Management Bureau, Florida Forest Service, Florida Department of Agriculture and Consumer Services, 3125 Conner Boulevard, I-255, Tallahassee, FL 32399 USA; 5 Japan Wildlife Research Center, Ogasawara Division, Okumura, Chichijima, Ogasawara, Tokyo, 100-2101 Japan; 6 United Center for Advanced Research and Translational Medicine, Graduate School of Medicine, Tohoku University, 2-1 Seiryo, Aoba, Sendai, 980-8575 Japan

## Abstract

Invaded species often can rapidly expand and establish in novel environments through adaptive evolution, resulting in devastating effects on native communities. However, it is unclear if genetic variation at whole-genomic levels is actually reduced in the introduced populations and which genetic changes have occurred responding to adaptation to new environments. In the 1960s, *Anolis carolinensis* was introduced onto one of the Ogasawara Islands, Japan, and subsequently expanded its range rapidly throughout two of the islands. Morphological comparison showed that lower hindlimb length in the introduced populations tended to be longer than those in its native Florida populations. Using re-sequenced whole genomic data, we estimated that the effective population size at the time of introduction was actually small (less than 50). We also inferred putative genomic regions subject to natural selection after this introduction event using SweeD and a method based on Tajima’s D, *π* and *F*_*ST*_. Five candidate genes that were potentially subject to selection were estimated by both methods. The results suggest that there were standing variations that could potentially contribute to adaptation to nonnative environments despite the founder population being small.

## Introduction

It has been recognized that evolutionary change is an important process in biological invasions, and many studies have reported evidence of rapid evolution of invasive species^1–5^. Furthermore, the success of an invasion might depend on adaptation to novel environments during the course of range expansion^6^. Several important evolutionary questions after a species invades a new area should be considered. First, the number of individuals (i.e., propagule pressure^7^) is often so small that low genetic diversity due to founder events could limit the species’ success and subsequent evolution^1^, however in many cases introduced species successfully expand their ranges by adapting to new environments. Several studies have suggested that repeated introductions into the nonnative population could provide the necessary genetic diversity for adaptive evolution^7–12^, but in some cases evolutionary changes after the initial introduction could still occur despite reduced genetic diversity of the populations^13^. A recent review^14^ concluded that many invaded species either have genetic diversity similar to or even greater than natives or do not face adaptive evolution to expand in the invaded area. These authors claimed that the genetic diversity in a small set of neutral genetic markers is not reflected in the genetic variation of ecologically relevant traits. Indeed, we are unaware of any study that estimates genetic variation of the introduced populations at the whole genomic level and reveals how such evolution was possible with reduced genetic variation in introduced population. Second, many studies on the evolution of invaded populations have examined evolutionary phenotypic changes^15–18^, but little is known about which genetic changes have occurred in response to adaptation to new environments.

The green anole, *Anolis carolinensis* (Sauria: Dactyloidae) is a lizard species native to the southeastern United States^19^. In addition to natural expansion within the United States, this species has been introduced to many areas around the world, including oceanic islands such as Taiwan, Hawaii, Guam, and some islands in Japan, where it has caused great impact on the native ecosystem by preying upon native animals^20–24^. *Anolis carolinensis* mainly inhabits trunks, branches and leaves on trees^25^. A recent study showed that native *A*. *carolinensis* on small artificial islands in Florida adapted and evolved larger toepads corresponding to the intentional introduction of *A. sagrei* within only 20 generations^26^. This suggests that *A. carolinensis* has the potential for rapid evolution responding to new environments over a short period of time.

**Figure 1:**
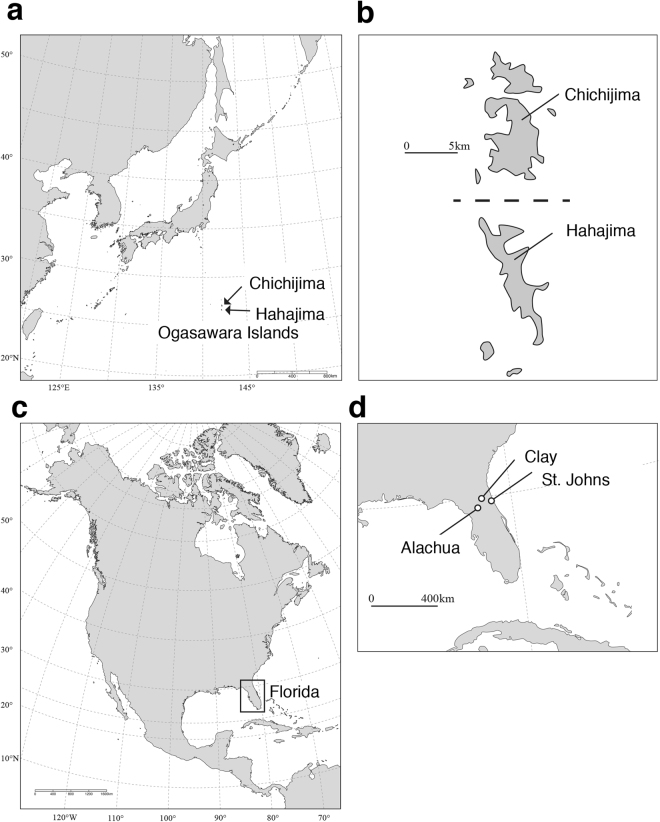
Sampling location of the green anole (*Anolis carolinensis*) in this study. (**a**) and (**b**) indicate the location of Chichijima and Hahajima Island in the Ogasawara archipelago, Japan. (**c**) and (**d**) indicate the location and the sampling sites in Florida, USA. The maps were redrawn from CC-BY open access allowed maps (DEX WEB: http://www.dex.ne.jp/download/map/) using Adobe Illustrator CS6 (ver 16.0.4, Serial 1543-0405-8063-3771-4051-0742).

The Ogasawara Islands (Bonin) consist of more than 30 small islands, and are located about 1,000 km south of the mainland of Japan (Fig. 1). The Ogasawara Islands were inscribed as a natural World Heritage Site in 2011 because many endemic species have been documented. *Anolis carolinensis* was introduced to Chichijima, one of the islands in the Ogasawara archipelago, in the 1960s, and it increased its population size after a lag-time phase^27^. Afterwards, it was introduced into the Hahajima islands in the 1980s from the Chichijima islands, and it has since expanded its range within these islands as well. This nonnative species is now believed to be responsible for local extirpation and disruption of the native ecosystem^28–30^. It is thought that *A. carolinensis* was introduced by the escape of pet animals and/or hitchhiking on transports of U.S. military forces^27^. Although the native range of the source population and the introduction pathway are uncertain, phylogenetic analysis suggests two mitochondrial DNA haplotypes in introduced Ogasawara populations, which are most closely related to the coastal region in Louisiana eastward to northern Florida^31,32^.

In this paper, we perform population genomic analyses in *A. carolinensis*. First, we re-sequenced the whole genome of individuals in the two introduced Ogasawara populations and in their native parental population in Florida. Next, we infer the demography of these populations, incorporating the founder population size and the rate of population expansion in the Ogasawara Islands. Finally, given the inferred demography, we infer candidate genes that may have been subject to positive selection in the introduced populations. Our purpose is not to determine the genes responsible for the adaptation of the invaded lizards, but estimate candidate genes and suggest a possible scenario for future studies.

## Materials and Methods

### Sample Collection

*Anolis carolinensis* was collected in Ogasawara archipelago Chichijima (*n* = 13) and Hahajima (*n* = 13) islands during 2011, and in northern Florida (*n* = 15) in 2010 (Fig. 1). Among these, 8 individuals were used for genome re-sequences for each population (Supplementary Table S1 and S2). We checked the haplotype of mitochondrial DNA (ND2) of 20 individuals (Chichijima, *n* = 8, Hahajima, *n* = 6; northern Florida, *n* = 6). Note that sequences of ND2 for 4 out of 24 individuals were not detected by genome resequencing, and therefore we used only 20 individuals. Then we checked the position of these haplotypes in Bayesian inference phylogeny of 398 individuals of *A. carolinensis* (see ref.^33^ for details on the method for constructing phylogeny). The haplotypes of all the localities of the northern Florida and Ogasawara individuals were clustered into the “Gulf Coast/Inland” clade defined in Campbell-Staton, *et al*.^31^ (see supplementary Fig. S1). This can justify that in modeling, the Florida population can be considered as the parental population of the introduced Ogasawara populations (see also the Discussion).

Our samples in the Ogasawara Islands were obtained from the collection of the Japan Wildlife Research Center. All the samples used were obtained from ethanol preserved dead body so that we need not get permit number from Tohoku University, but all animal treatments were performed according to the guidelines of the Animal Care and Use Committee of Tohoku University, Miyagi Prefecture, Japan.

### Analysis

Only adult males were used for morphological measurements (Chichijima, *n* = 13; Hahajima, *n* = 13). The following morphological variables were measured for each individual according to the established ecological significance of these traits in genus *Anolis* ^34^: (1) snout–vent length (SVL) from the tip of the snout to the anterior end of the cloaca, which is used as the overall body size, (2) head length (HL) from the back of the parietal to the tip of the snout, (3) head width (HW), the distance across the head measured at the anterior end of the ear, (4) lower hindlimb length (LHL) from the apex of the knee to the center of the ankle. Morphological variables of Florida populations were obtained from a previous study (Alachua *n* = 5, Clay *n* = 4, Saint Johns *n* = 6)^35^. ImageJ64 v.1.47n software^36^ was used for all the measurements.

Residual values of the regressions of the log10-transformed morphological data against log10-transformed SVL were calculated to remove the effects of body size. A principal components analysis (PCA) was conducted using these residual values. For statistical analysis, we used Tukey’s honestly significant difference (HSD) test.

### DNA Extraction and Genome Sequence

Whole genome re-sequencing was conducted for 24 individuals (eight individuals from each of the three populations, Chichijima, Hahajima, and Florida). After measurements of morphological variables were taken, muscle tissue was preserved in 95% ethanol, stored at −20C°, and then used for DNA extraction using Qiagen genomic tip 100/g. The reference genome was used for mapping reads obtained from the re-sequencing experiment. The insert sizes and the number of reads for each sample are shown in Supplementary Table S3. Whole genome reference sequences^37^ were used for the genome resequencing. The DNA libraries were clonally amplified on a flow cell and sequenced on HiSeq. 2500 (HiSeq Control Software v2.0.12.0, Illumina) with 101-mer paired-end sequences. Image analysis and base calling were performed using Real-Time Analysis Software (v1.17.21.3, illumina). DNA genome sequence data for 24 *A. carolinensis* individuals have been deposited in DDBJ under accession numbers DRA004461 to DRA004485.

### Read Quality Control

To remove low quality reads, we checked the read quality by applying software FastQC v0.10.1^38^. We excluded low quality reads (more than 30% of nucleotides with quality value less than 30) and adaptor sequences by FASTX-toolkit v0.0.13 (http://hannonlab.cshl.edu/fastx_toolkit). Furthermore, we trimmed 3 bp from the 5′ and 3′ ends of sequences with a quality value of more than 20 for each read, because their read quality was relatively low. Note that we did not use trimmed reads when their length was less than 20 bp. These filtered reads were used for further analysis.

### Mapping and SNP Detection

Before read mapping, the insert size of each read was checked using picard-tools v1.114 (http://broadinstitute.github.io/picard and then each read was mapped to the *A. carolinensis* reference genome (from Ensembl release 80, download from: http://www.ensembl.org/) using bowtie v1.0.1. We performed SNP calling using SAMtools mpileup with default parameters v0.1.18^39^. Biallelic SNPs with a quality score >10 were used for our analyses. SNPs in high read coverage regions (>100) were excluded. SNP deviating from Hardy–Weinberg Equilibrium (P < 0.01) were excluded.

### Population structure analysis

To assess population structure, we used ADMIXTURE v1.23^40^ implementing a model-based maximum likelihood clustering algorithm. We tested the number of ancestral populations (*K*) from 2 to 5.

#### Inference of Demographic History

We assumed the demographic model of the two populations following ref.^27^ (Supplementary Fig. S2). *Anolis carolinensis* was assumed to be introduced directly from USA to Chichijima Island. Although the population of the Chichijima Island could have been introduced from North America via Guam^41^, we did not take that possibility into consideration because the two introduction events occurred during a very short period of time (1950s into Guam and 1960s into Ogasawara^42^); the model is described with four demographic parameters (Supplementary Fig. S2): the native population size (*N*_1_), the time of divergence between introduced and native population (*T* generations), the initial population size of introduced population (*N*_2b_), and the present population size of the introduced population (*N*_2f_). An exponential change in population size is assumed in the introduced population. We originally constructed a model with migration between the two populations, but higher likelihood was produced from lower migration rates. Moreover, their effect on likelihood was relatively weak presumably because of the very short time since the invasion. Thus, we excluded migration from the model for simplicity.

Parameters were estimated with the software δaδi v1.7.0^43^, which uses a diffusion method and joint allele frequency spectra (AFS) to evaluate the likelihood of the model. First, we calculated the observed joint AFS of the Chichijima and Florida populations. The number of total sites used in the analysis (only non-coding sites) was 1,212,784. The number of polymorphic sites in Chichijima and Florida were 506,335 and 1,041,548, respectively. *T* was fixed to 50 according to the record. Mutation rate was assumed to fall between 10^−9^ and 10^−7^ per site per generation^44–46^. We conducted the same analysis with three different mutation rates (10^−9^, 10^−8^, and 10^−7^), and obtained very similar estimates and expected AFS, which generated almost identical null distributions of summary statistics for the downstream analyses. Here we report only the results with 10^−8^. *N*_1_ was calculated from the observed value of nucleotide diversity 14.0 per 10 kb and mutation rate, and set to 35,000. Then, the combination of *N*_2b_ and *N*_2f_ to maximize the likelihood was searched. The parameter ranges of *N*_2b_ and *N*_2f_ were 2–1,000 and 35–35,000, respectively. Note that this parameter space includes population decline after the introduction (*N*_2b_ > *N*_2f_).

#### Inference of candidate regions for selection

Using the inferred demographic parameters, coalescent simulations with ms^47^ were performed 1,000,000 times to generate the null distributions of summary statistics: Tajima’s *D*, nucleotide diversity (*π*) and *F*_*ST*_^48^. We obtained the observed value of those summary statistics by performing a sliding window analysis on Ogasawara population genomes with 10 kb window size and 5 kb sliding size. P < 0.05 (one-tailed test) was used as a criterion to find potential regions evolving under directional selection in the invaded populations. We also examined the analysis using other window sizes (window size [ws] = 20 kb, sliding size [ss] = 10 kb; ws = 5 kb, ss = 2.5 kb), but the trends of our results did not change. We applied the simulation results from Chichijima for Hahajima analysis too, because the two populations were classified into the same cluster in ADMIXTURE analysis (Supplementary Fig. S3). The same analysis with δaδi and ms was performed by pooling the genomes of both Chichijima and Hahajima islands, and almost identical null distributions and threshold values were obtained. Thus, the same demographic parameters could be applied to the Hahajima population. Genomic regions with negative Tajima’s *D*, low *π* and positive *F*_*ST*_ were considered to be candidate genes that had been potentially subject to positive selection and had diverged after the introduction to the Ogasawara Islands.

In addition, to infer the selective regions from the genome in Ogasawara populations, we performed an analysis using SweeD software (version 3.2.12)^49^, which implements a composite likelihood ratio test to detect complete selective sweeps using Site Frequency Spectrum (SFS) patterns of SNPs. SweeD analysis using SNSs of Chichijima and Hahajima populations was conducted with default parameter settings except the number of windows (108164, which was calculated as the genome length[1,081,644,591]/ the window size used in the above sliding window size[10 kb]).

### GO analysis

To assess enrichment of gene ontology terms of candidate genes, we performed gene ontology (GO) analysis. For analysis, we build the custom GO annotations for *A. carolinensis* based on chicken, mouse and human GO databases, deriving each orthologous relationship from the Ensembl database (http://www.ensembl.org). The GO identifier (ID) biological process was used. We calculated the *P* value for each GO ID by comparison of the number of observed GO IDs with that of expected GO IDs based on a hypergeometric distribution. The estimated *P* values were adjusted by Bonferroni correction.

## Results

**Figure 2:**
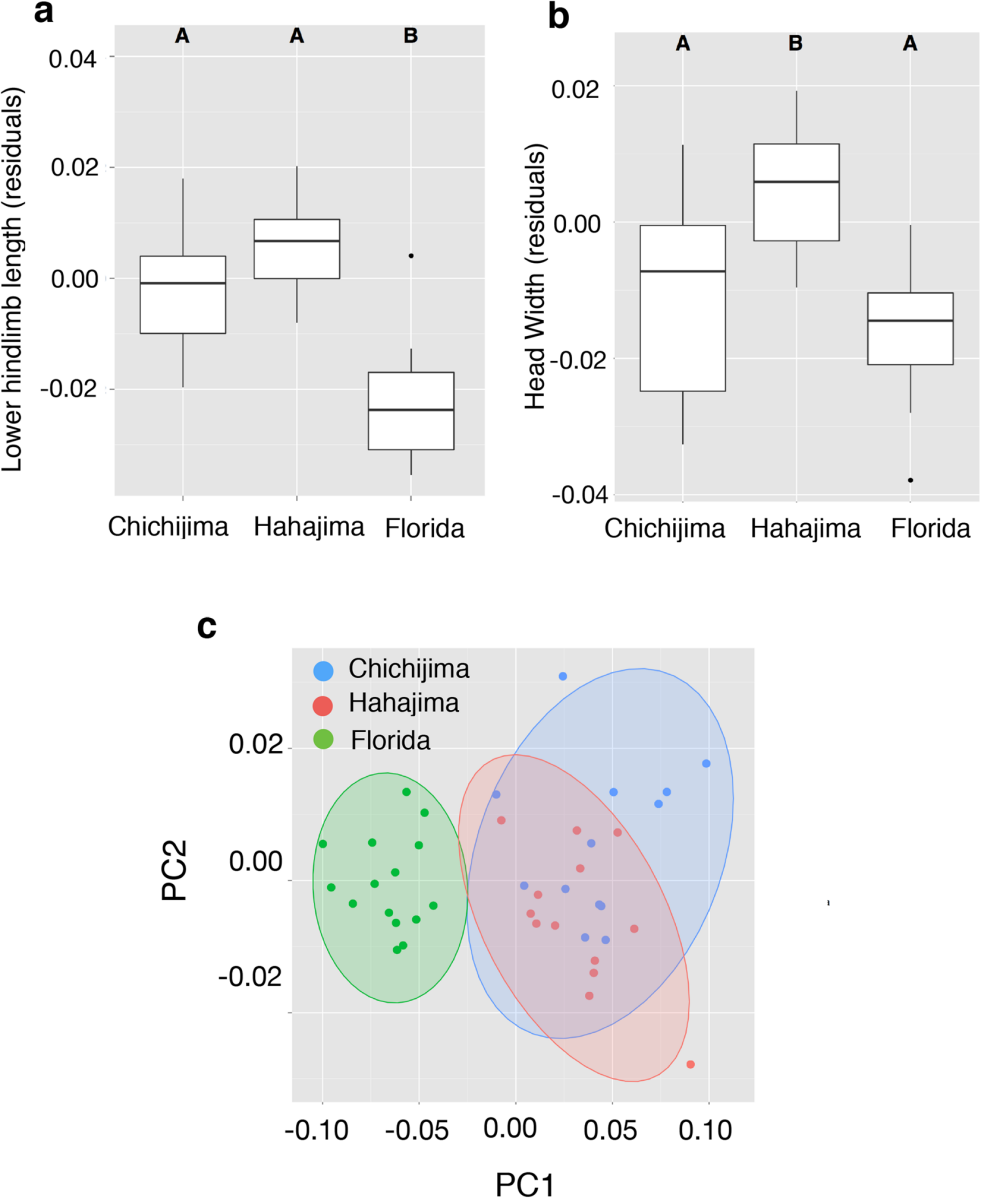
Morphological traits among the introduced (Chichijima and Hahajima) and native populations (Florida) of the green anole (*Anolis carolinensis*). (**a**) Residual values of regression of lower hindlimb length on snout-vent-length (SVL). A, B; different character indicates significantly different means. Tukey’s HSD test, Chichijima vs. Florida, *P* = 0.0003, Hahajima vs. Florida *P* < 0.0005. (**b**) Residual values of regression between head width on SVL. A, B; different character indicates significantly different means. Tukey’s HSD test, Hahajima vs. Florida *P* < 0.0005. (**c**) Principal components analysis (PCA) of morphological measurements. Only PC1 and PC2 axes are shown. Lower hindlimb length largely contributed to PC1, while head length did to PC2 (Table S1). Each point indicates Chichijima (Blue), Hahajima (Red) and Florida (Green) samples.

We compared morphological characters among the introduced and native populations. Lower hindlimb length (LHL) in the introduced populations was longer than in the native Florida population (Fig. 2a, Tukey’s HSD test, Chichijima vs. Florida, *P* = 0.0003, Hahajima vs. Florida *P* < 0.0005). Head width in Hahajima is significantly longer than in Chichijima and Florida (Fig. 2b, Tukey’s HSD test, Hahajima vs. Florida *P* < 0.0005), although there were no significant differences in head length among the populations (Tukey’s HSD test, Hahajima vs. Florida *P* = 0.9997). Principal component analysis (PCA) also showed the same trends (Fig. 2c, Supplementary Table S4).

We sequenced a total of 24 individuals, and re-sequencing results are summarized in Table 1 and Supplementary Table S2. The mean percentage of mapped reads was 97.65%. There were massive reads mapped on extremely high coverage regions where there were repetitive sequences, thus these were excluded from our analyses. After filtering out reads within high coverage regions (read depth >1,000), the mean frequency of mapped reads was 50.24%. The average coverages in Chichijia, Hahajima and Florida individuals were 7.05, 5.85, and 7.97, respectively. There are no differentiations in the sequencing quality among populations (Tukey’s honestly significant difference (HSD) test, Supplementary Table S5). The number of polymorphic sites and the mean nucleotide diversity (π) in the introduced populations were about 53% and 60% of those of the native population, respectively (Table 1). Mean Tajima’s *D* in the Chichijima population was 0.949, Hahajima population was 0.988, but that in the native population was 0.120 (Table 1). Population genetic structure estimated by ADMIXTURE shows that all of the examined individuals in the Chichijima and Hahajima populations were classified as the same genetic assignments, while the individuals of native Florida were different (although a few individuals had the same genetic components as the individuals of Chichijima and Hahajima populations) (Supplementary Fig. S3). This suggests that the introduced populations shared the same genetic cluster originating from a part of the genetic clusters of the native Florida population.

**Table 1:** Summary genetic statistics of introduced and native populations of the green anole (*Anolis carolinensis*).

Population	Introduced/Native	Total sites	Polymorphic sites	nucleotide diversity (πper 10 kb)	Watterson’s theta (θ)	Tajima’s *D*	*F*_*ST*_
Chichijima	Introduced	1,212,784	924,079	9.0 (5.80 × 10^−8^)	9.9090	0.949 (1.178)	0.106 (0.006)
Hahajima	Introduced		928,130	9.0 (5.80 × 10^−8^)	9.8185	0.988 (1.035)	0.108 (0.005)
Florida	Native		1,123,266	14.0 (5.80 × 10^−8^)	19.45643	0.12 (0.301)	

Total sites indicate the number of sites that could be assembled for all the 24 individuals. *F*_*ST*_ indicates genetic distance between introduced and native populations. The values in parentheses indicate variance across all the windows (window size = 10 kb).

We inferred the demographic history modeled as shown in Supplementary Fig. S2. The estimated values of the effective size at introduction (*N*_2b_) and at the present time (*N*_2f_) were 14.4 and 1290, respectively (log likelihood values = −124,404.4585, Supplementary Figs. S2 and S4d). Although these two parameters are confounding (Supplementary Fig. S4), the distribution of Tajima’s *D*, *F*_*ST*_ and π generated by coalescent simulations (Supplementary Fig. S5) did not greatly change using other plausible combinations of the estimated values. Even when other plausible combinations of estimates *N*_2b_ and *N*_2f_ (e.g., *N*_2b_ ≈ 45 and *N*_2f_ = 100) were considered, the number of originally introduced individuals should be small (less than 50).

**Figure 3:**
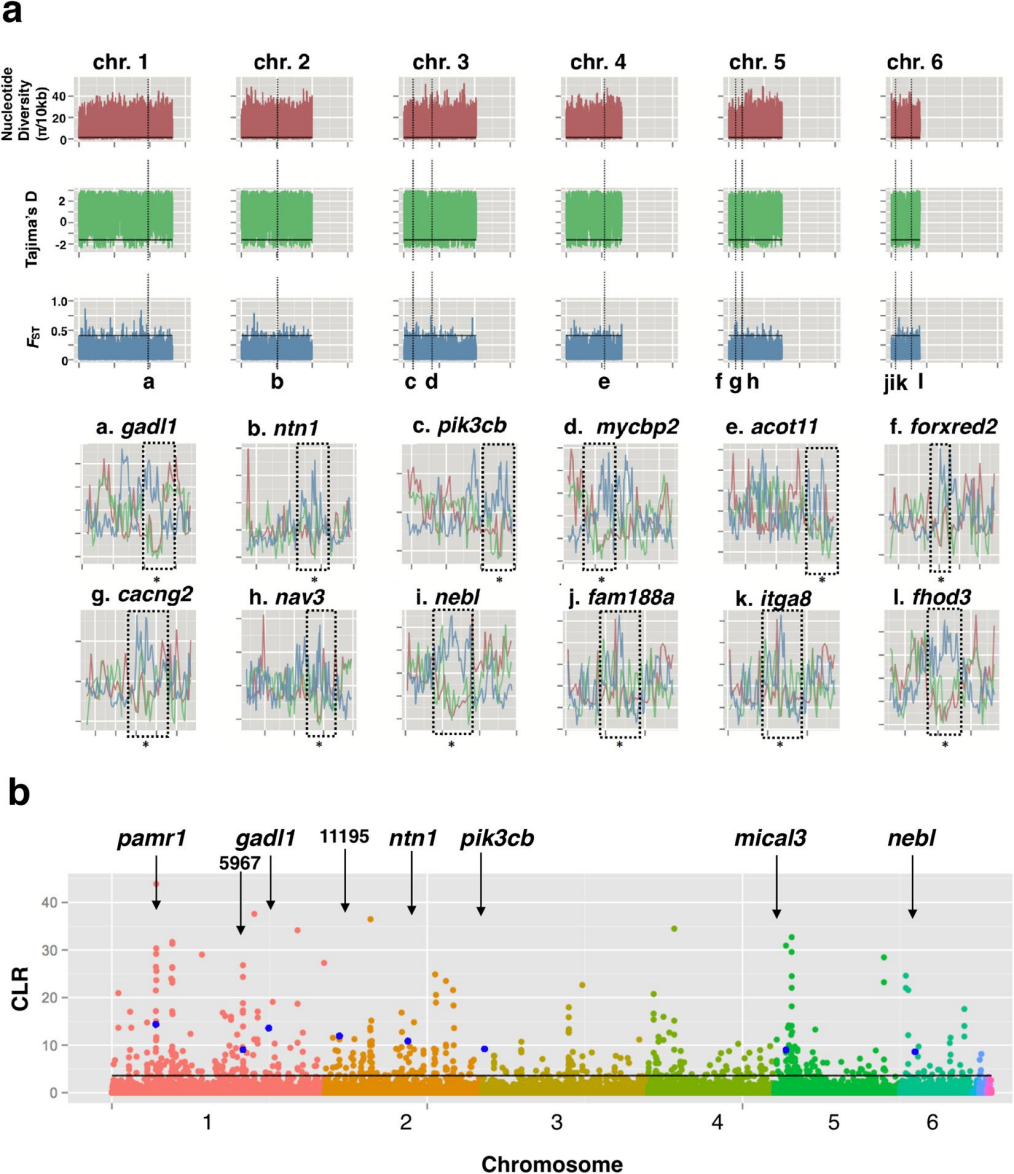
The estimation results using whole-genome sequences of the green anole (*Anolis carolinensis*). (**a**) Genome-wide sliding window analysis (window size = 10 kb, sliding size = 5 kb) of Tajima’s *D, π* and *F*_*ST*_ in the introduced population. The regions of chromosomes 1–6 are shown. The horizontal dashed line indicates the threshold at *P* < 0.05. *P* values were determined based on the null distribution produced by the coalescent simulation. The lower figure shows 12 genomic regions with significantly lower Tajima’s *D*, *π* and higher *F*_*ST*_ (*P* < 0.05) detected both in the Chichijima and Hahajima populations. Dashed boxes indicate ±50 Kbp flanking regions of the sites. (**b**) Plot of the composite likelihood ratio cutoff value for the 0.1% outliner regions estimated using SweeD. Eight candidate genes were shown.

Based on the null distributions by the coalescent simulations (Supplementary Fig. S5), we detected genes in which all the values of Tajima’s *D*, *F*_*ST*_ and π were significant (P < 0.05). Seventeen and 15 candidate genes that might be subject to positive selection were detected in the Chichijima and Hahajima populations, respectively (Table 2 and Fig. 3a). Five genes were only detected in the Chichijima populations (Table 2), and three genes were only detected in the Hahajima populations (Table 2). Twelve candidate genes were shared between the Chichijima and Hahajima populations (Table 2, Fig. 3a). When the null distributions were generated by pooling the genomes of both Chichijima and Hahajima islands, almost identical threshold values were obtained, and only one gene (*capn13*) among 20 genes (Table 2) was excluded as non-significant. SweeD detected eight candidate genes, and five of them were also detected by the method based on the values of Tajima’s *D*, *F*_*ST*_ and π (Table 2 and Fig. 3b). The allele frequencies of these candidate genes in the Florida populations were usually less than 0.2, while those in the Ogasawara populations ranged from 0.7 to 1.0 (Supplementary Table S6).

**Table 2:** Inferred candidate genes that had been potentially subject to selection in the introduced populations of the green anole (*Anolis carolinensis*).

Chrom.	Gene	Ensembl ID	Tajima’s *D*		π		*F*_*ST*_		*Related GO term name*
			Chichijima	Hahajima	Chichijima	Hahajima	Chichijima	Hahajima	
(**a**)									
1	***gadl1*** *	14308	−1.78	−1.70	0.59	1.13	0.45	0.43	metabolic process
1	unknown*	5967	−**2.32**	−	1.13	—	0.43	—	calcium ion binding
1	*capn13*	7376	−1.77	—	6.43	—	0.45	—	proteolysis
2	***ntn1*** *	17710	−1.75	−1.71	0.85	1.00	0.41	0.45	axonogenesis
2	***itpr1***	9987	−2.12	—	0.66	—	0.42	—	voluntary musculoskeletal movement
2	*cntn6*	10425	−1.77	—	0.98	—	**0.56**	—	protein binding
2	*acvr1b*	16836	—	−2.10	—	1.39	—	0.42	embryo development
3	***pik3cb*** *	2884	−2.10	−1.93	**0.25**	1.18	0.43	0.44	phosphorylation
3	***mycbp2***	646	−1.72	−2.03	1.30	1.33	0.46	0.43	motor neuron axon guidance
3	*wwtr1*	7527	−1.86	—	1.34	—	0.44	—	osteoblast differentiation
4	***acot11***	14353	−1.78	−1.71	1.36	1.06	0.43	0.44	response to temperature stimulus
5	***foxred2***	16347	−1.93	−2.06	1.24	1.17	0.46	0.41	oxidation-reduction process
5	***cacng2***	16285	−1.83	−1.83	0.68	0.69	0.43	0.44	transmission of nerve impulse
5	***nav3***	14617	−1.70	−1.86	1.13	1.35	0.45	0.44	ATP binding
5	***scfd2***	10600	—	−2.13	—	1.02	—	0.42	vesicle docking involved in exocytosis
6	nebl2*	9235	−2.16	−2.15	1.02	1.13	0.42	0.42	cardiac muscle thin filament assembly
6	***fam188a***	16625	−2.00	−1.91	0.78	**0.25**	0.55	0.51	calcium ion binding
6	***itga8***	16491	−1.80	−1.83	0.50	0.45	0.41	0.42	smooth muscle tissue development
6	***fhod3***	6717	−1.85	−1.76	**0.23**	0.50	0.41	0.42	actin filament organization
6	Unknown	3820	—	−1.74	—	1.25	—	0.50	
**(b)**									
**Chromosome**	**Gene**	**Ensembl ID**	**Composite Likelihood rate (CKR)**	***Related GO term name***					
1	*pamr1*	4544	14.35	proteolysis					
1	unknown*	5967	9.04	calcium ion binding					
1	gadl1*	14308	13.58	metabolic process					
2	unknown	11195	11.92	cell adhesion					
2	ntn1*	17710	10.85	axonogenesis					
3	pik3cb*	2884	9.19	phosphorylation					
5	*mical3*	12545	8.99	Protein biding					
6	nebl*	9235	8.59	cardiac muscle thin filament assembly					

(a) The genes inferred based on Tajima’s *D*, π and *F*_*ST*_. Genes with *P* < 0.05 are shown. We considered genes where all Tajima’s *D*, πand *F*_*ST*_ values were significant (*P* < 0.05) based on the null distribution from the simulations. *P* values were estimated based on the null distributions generated by coalescent simulations. Genes inferred from both Chichijima and Hahajima are shown in bold. Bold values indicate *P* < 0.01. (b) The genes inferred using SweeD software. SNP data from both Chichijima and Hahajima were used. Asterisk indicates the genes detected in both methods. The minimum number of SNPs in a window including detected genes were 44,448 and 276,607 in the method based on Tajima’s *D*, π and *F*_*ST*_ on and SweeD, respectively.

We performed Gene Ontology (GO) analysis using candidate genes detected by the method based on the values of Tajima’s *D*, *F*_*ST*_ and π and those detected by SweeD. There were no significantly enriched GO functional categories for these genes.

## Discussion

*Anolis carolinensis* was introduced to one of the Ogasawara Islands in the 1960s, and it subsequently increased its population size. Our study estimated the effective size of the introduced population at the introduction and detected putative genomic regions subject to natural selection after this introduction event using 24 individuals of re-sequenced genomic data. We also compared morphological data between the introduced populations and the native Florida populations. The results suggest that the effective size at the time of introduction was actually small and we detected twelve candidate genes for selection in both the Ogasawara Islands.

In this study, we assumed that our samples from northern Florida represent the source populations of invaded individuals in Ogasawara. As described in the Methods, the mtDNA haplotypes (ND2) of Ogasawara individuals were clustered into the “Gulf Coast/Inland” clade^31^. The Florida samples were scattered across the branches in the “Gulf Coast/Inland” clade. At the same time, the distribution of Tajima’s *D* obtained from sliding windows on Florida genomes was finely bell-shape and centered on zero, which indicates a non-structured and stable population (Supplementary Fig. S6). We thus assumed that our Florida individuals could represent the putative ancestral population, the Gulf Coast/Inland clade. In the present study, positive *F*_*ST*_s were considered to be one of the criteria detecting candidate genes that had been subject to positive selection and had diverged after the introduction into the Ogasawara Islands. Since we assumed that lizards were transported from North America to the Ogasawara islands during a short period of time, the detected genes might be considered to have diverged after introduction to the Ogasawara Islands. Based on the records of the time of introduction in Guam and Ogasawara, we could assume the introduction into Ogasawara from North America had occurred within 10 years.

The estimated effective population size at the introduction was small (less than 50). This indicates that a small founder population with reduced genetic variation could have made such evolutionary responses during 50 generations. Many invaded species have experienced a bottleneck, which reduced genetic variation and suggests that they are supposed to have little evolutionary potential. Estoup *et al*.^14^ concluded that many invaded species either have genetic diversity similar to or even greater than native species. The present results indicate that the effective population size (i.e., genetic variation) was small at the time of introduction. Although we cannot exclude the possibility that the genetic modifications after the introduction to Chichijima result from new mutations during population expansion, the positively skewed Tajima’s *D* distribution suggests that there were some initial standing genetic variations in introduced individuals that could potentially contribute to adaptation to nonnative environments and rapid expansion in the Ogasawara Islands.

Morphological comparison showed that lower hindlimb length in the introduced populations was significantly longer than in native Florida populations, although the sample size was not large enough for firm conclusions and it is unknown whether or not these differences were due to genetic components. Hindlimb length is one of the known characters that influence adaptation to structural habitats in *Anolis* lizards^34^. A long hindlimb is efficient for effectively moving around trunk and ground microhabitats by promoting running performance^34,50^. *Anolis carolinensis* usually inhabits trunk to tree crown, but it moves closer to the ground when a competitor is absent. According to reports of *A. carolinensis* in Ogasawara (Chichijima and Hahajima)^51^ and United States (three Florida and one Georgia population)^52^, perch height (PH) and perch diameter (PD) used by the anoles in Ogasawara (PH = 73.3 mm and PD = 60.3 mm, the mean values of male and females in Chichijima and Hahajima^51^) were lower and larger than those in Florida and Georgia (PH = 92.9 mm and PD = 37.1 mm, the mean values of male and females in four populations, ref.^52^), respectively. Although the morphology measures taken in Florida individuals were not necessarily those of the source population of the invaded ones, anoles in Ogasawara might use much lower microhabitats and thicker twigs or trunks than those in the United States. It has been reported that there is a positive correlation between relative hindlimb length and mean perch diameter^53^. Therefore, long hindlimb length observed in these introduced populations might be related to adaptive changes to microhabitat uses.

Based on *F*_ST_, Tajima’s *D* and nucleotide diversity, twelve genes were inferred as candidate genes that had been potentially subject to selection in both Chichijima and Hahajima populations. SweeD based on site frequency spectrum inferred eight genes as candidate genes for selection. Among these genes, four genes (PIK3CB, NEBL, GADL1, NTN1) were inferred by both methods, A gene (unknown genes, Ensembl ID = 5967) was inferred by SweeD and it was also detected in the Ogasawara Islands based on the method used in *F*_ST_, Tajima’s *D* and nucleotide diversity values. In the present study, we could use genome sequences of only 24 individuals. Owing to small sample sizes, we could not detect genes experiencing weak selection, and these candidate genes might be erroneously detected due to the larger effect of genetic drift. Although we could not rule out the effect of genetic drift, the frequencies of the candidate genes were high only in the Ogasawara Islands, but these were low (<0.3) in native Florida populations so that these genes could increase in frequency after the introduction. In addition, we could not fully rule out the possibility that successfully invaded individuals had particular genotypes conferring invasive success.

Five candidate genes (*pik3cb*, *nebl*, *gadl1*, *ntn1*, and ENSACAG5967) were detected that might be subject to selection in the Ogasawara Islands using both the methods. There were no significantly enriched GO functional categories for these detected candidate genes. However, it is worth discussing the function of these genes, although we could not provide any convincing evidence for the relationships between these genes and their functional importance after the introduction event. Among the five candidate genes, two genes (*nebl* and *gadl1*) have functions in muscle development and contraction, and two other genes (*ntn1* and *pik3cb*) have a function in the metabolic process. In addition, other candidate genes detected only by one of the two methods also have functions in muscle development and muscle contraction (*fhod3*, *fam188a*, *itga8*, *cacng2* and *pamr1* and *mical3*), and the metabolic process (*acot11*). These genes related to muscle development and muscle contraction might be related to the evolution of longer hindlimb length and related changes in movements and microhabitat use in the introduced populations. Candidate genes, such as *ntn1*, *acot11* and *pik3cb*, are related to changes in the metabolic process. All of the three genes have a function related to insulin resistance and obesity in mammals^54–56^. These selected genes imply that the evolutionary changes of insulin regulation might help adapting to changes in food items and diet composition after the introduction.

The impact of *A. carolinensis* on native communities has become a crucial ecological issue in the Ogasawara Islands. A large number of endemic insect species has gone extinct by the predatory impact of *A. carolinensis* ^57,58^. Evolutionary adaptation to changes in food items in the Ogasawara Islands might have accelerated the predatory damages on native species. Our study suggests that *A. carolinensis* has expanded its populations and changed its morphology associated with shifting habitat use. The results suggest a hypothesis that genes associated with niche shift (changes in microhabitat and feeding habit) have been subject to selection after the introduction event. These evolutionary changes during such a short period of time could occur since the initial standing variations in introduced individuals might be responsible for adaptation to nonnative environments despite the founder population being small. However, more robust genomic analyses such as haplotype-based inferences using larger sample sizes are necessary to provide firm conclusions.

## Electronic supplementary material

Supplemental Tables and Figures
