# Supplementary material for: Inferring evolutionary responses of Anolis carolinensis introduced into the Ogasawara archipelago using whole genome sequence data
Source: Sci Rep. 2017 Dec 21;7:18008. doi: 10.1038/s41598-017-17852-7 (PMC5740155; doi:10.1038/s41598-017-17852-7)
Supplement: Supplementary file 1 — Supplemental Tables and Figures [file 41598_2017_17852_MOESM1_ESM.doc]

**Supplement Tables and Figures**

**Inferring evolutionary responses of *Anolis carolinensis* introduced into Ogasawara archipelago using whole genome sequence data**

Satoshi Tamate^1†^, Watal M. Iwasaki^2†^, Kenneth L. Krysko^3^, Brian J. Camposano^4^, Hideaki Mori^5^, Ryo Funayama^6^, Keiko Nakayama^6^, Takashi Makino^1*^ and Masakado Kawata^1*^

^1^Department of Ecology and Evolutionary Biology, Graduate School of Life Sciences, Tohoku University, Aoba-ku, Sendai, 980-8578, Japan

^2^Department of Evolutionary Studies of Biosystems, SOKENDAI (The Graduate University for Advanced Studies), Hayama, Japan
^3^Division of Herpetology, Florida Museum of Natural History, 1659 Museum Road, University of Florida, Gainesville, FL 32611, USA
^4^Forest Management Bureau, Florida Forest Service, Florida Department of Agriculture and Consumer Services, 3125 Conner Boulevard, I-255, Tallahassee, FL  32399, USA
^5^Japan Wildlife Research Center, Ogasawara Division, Okumura, Chichijima, Ogasawara, Tokyo 100-2101, Japan
^6^ United Center for Advanced Research and Translational Medicine, Graduate School of Medicine, Tohoku University, 2-1 Seiryo, Aoba, Sendai 980-8575, Japan

^†^Equal contributors

*Corresponding authors

Running title: Evolution of an invaded species

**Supplement Tables**

**Table S1. Summary information of the sampling sites and longitude/latitude of the green anole (*Anolis carolinensis*)**.

| **Sample name** | **Location** | **Lat/long** |
| --- | --- | --- |
| C0001 | Chichijima | 27.358, 142,12,51 |
| C0002 |  | 27.358, 142,12,51 |
| C0003 |  | 27.358, 142,12,51 |
| C0004 |  | 27.358, 142,12,51 |
| C0005 |  | 27.358, 142,12,51 |
| C0006 |  | 27.358, 142,12,51 |
| C0007 |  | 27.358, 142,12,51 |
| C0008 |  | 27.358, 142,12,51 |
| H0001 | Hahajima | 26.3941, 142.926 |
| H0002 |  | 26.3941, 142.926 |
| H0003 |  | 26.3941, 142.926 |
| H0004 |  | 26.3941, 142.926 |
| H0005 |  | 26.3941, 142.926 |
| H0006 |  | 26.3941, 142.926 |
| H0007 |  | 26.3941, 142.926 |
| H0008 |  | 26.3941, 142.926 |
| F0044 | Florida: Alachua County | 29.6945, -82.3545 |
| F0045 |  | 29.6945, -82.3545 |
| F0046 | Florida: Clay County | 30.1286, -81.9648 |
| F0047 |  | 30.1286, -81.9648 |
| F0048 |  | 30.0230, -81.3283 |
| F0050 | Florida: Saint Johns County | 30.0230, -81.3283 |
| F0051 |  | 30.0230, -81.3283 |
| F0052 |  | 30.0230, -81.3283 |

**Table S2. Genome mapping scores and coverage of each individual green anole (*Anolis. carolinensis*).**

| **Individual** | **Population** | **Median insert size** | **Total number of reads** | **Mapped reads** | **Mapped reads (%)** | **Total read length** | **Coverage** |
| --- | --- | --- | --- | --- | --- | --- | --- |
| C0001 | Chichijima | 147 | 104072405 | 54315941 | 52.19% | 7984443327 | 7.3818 |
| C0002 |  | 176 | 102966103 | 52457684 | 50.95% | 9232552384 | 8.5357 |
| C0003 |  | 177 | 79761248 | 41549602 | 52.09% | 7354279554 | 6.7992 |
| C0004 |  | 184 | 79247114 | 40871316 | 51.57% | 7520322144 | 6.9527 |
| C0005 |  | 175 | 83570226 | 42310186 | 50.63% | 7404282550 | 6.8454 |
| C0006 |  | 177 | 77994842 | 38712423 | 49.63% | 6852098871 | 6.3349 |
| C0007 |  | 178 | 84309053 | 42468592 | 50.37% | 7559409376 | 6.9888 |
| C0008 |  | 176 | 82632130 | 40283944 | 48.75% | 7089974144 | 6.5548 |
| Chichijima mean |  | 174 | 86819140 | 44121211 | 50.77% | 7624670294 | 7.0491 |
| H0001 | Hahajima | 161 | 88109395 | 46149135 | 52.38% | 7430010735 | 6.8692 |
| H0002 |  | 177 | 61375175 | 30670329 | 49.97% | 5428648233 | 5.0189 |
| H0003 |  | 179 | 54618283 | 27287169 | 49.96% | 4884403251 | 4.5157 |
| H0004 |  | 177 | 67868601 | 34410268 | 50.70% | 6090617436 | 5.6309 |
| H0005 |  | 183 | 56861714 | 28962138 | 50.93% | 5300071254 | 4.9000 |
| H0006 |  | 183 | 68395904 | 35787572 | 52.32% | 6549125676 | 6.0548 |
| H0007 |  | 178 | 78089326 | 41016545 | 52.53% | 7300945010 | 6.7499 |
| H0008 |  | 182 | 80674427 | 42132590 | 52.23% | 7668131380 | 7.0893 |
| Hahajima mean |  | 178 | 69499103 | 35801968 | 51.38% | 6331494122 | 5.8536 |
| F0044 | Florida | 210 | 97861723 | 49781593 | 50.87% | 10454134530 | 9.6650 |
| F0045 |  | 180 | 105141777 | 44182846 | 42.02% | 7952912280 | 7.3526 |
| F0046 |  | 188 | 100940396 | 48323861 | 47.87% | 9084885868 | 8.3991 |
| F0047 |  | 170 | 111333943 | 53013148 | 47.62% | 9012235160 | 8.3320 |
| F0048 |  | 162 | 116042668 | 52601398 | 45.32% | 8521426476 | 7.8782 |
| F0050 |  | 199 | 92471115 | 47632034 | 51.51% | 9478774766 | 8.7633 |
| F0051 |  | 203 | 72214632 | 37395304 | 51.78% | 7591246712 | 7.0182 |
| F0052 |  | 200 | 66447038 | 34284990 | 51.60% | 6856998000 | 6.3394 |
| Florida mean |  | 189 | 95306662 | 45901897 | 48.57% | 8619076724 | 7.9685 |

**Table S3. Insert size and the number of reads.**

| Sample No. | Minimum insert size | Maximum insert size | Mean insert size | S.d. |  | Number of Reads |
| --- | --- | --- | --- | --- | --- | --- |
| C1 | 23 | 350 | 193 | 45 |  | 54,315,941 |
| C2 | 25 | 350 | 185 | 46 |  | 52,457,684 |
| C3 | 24 | 350 | 186 | 46 |  | 41,549,602 |
| C4 | 35 | 350 | 191 | 47 |  | 40,871,316 |
| C5 | 32 | 350 | 183 | 42 |  | 42,310,186 |
| C6 | 33 | 350 | 187 | 52 |  | 38,712,423 |
| C7 | 34 | 350 | 186 | 45 |  | 42,468,592 |
| C8 | 24 | 350 | 184 | 49 |  | 40,283,944 |
| average | 29 | 350 | 187 | 46 |  | 44,121,211 |
| H1 | 23 | 350 | 167 | 33 |  | 46,149,135 |
| H2 | 33 | 350 | 184 | 40 |  | 30,670,329 |
| H3 | 33 | 350 | 187 | 42 |  | 27,287,169 |
| H4 | 33 | 350 | 184 | 40 |  | 34,410,268 |
| H5 | 34 | 350 | 190 | 40 |  | 28,962,138 |
| H6 | 22 | 350 | 190 | 44 |  | 35,787,572 |
| H7 | 22 | 350 | 186 | 42 |  | 41,016,545 |
| H8 | 32 | 350 | 189 | 43 |  | 42,132,590 |
| average | 29 | 350 | 185 | 40 |  | 35,801,968 |
| F1 | 32 | 350 | 216 | 53 |  | 49,781,593 |
| F2 | 32 | 350 | 188 | 51 |  | 44,182,846 |
| F3 | 33 | 350 | 196 | 51 |  | 48,323,861 |
| F4 | 24 | 350 | 177 | 47 |  | 53,013,148 |
| F5 | 34 | 350 | 168 | 41 |  | 12,259,336 |
| F6 | 22 | 350 | 206 | 52 |  | 47,632,034 |
| F7 | 26 | 350 | 210 | 53 |  | 37,395,304 |
| F8 | 24 | 350 | 208 | 53 |  | 34,284,990 |
| average | 28 | 350 | 196 | 50 |  | 40,859,139 |

**Table S4. Loadings from a PCA for size-adjusted morphological variables of the green anole (*Anolis carolinensis***).

| **Morphological variable** | **PC1** | **PC2** | **PC3** |
| --- | --- | --- | --- |
| Head length (mm) | 0.113 | -0.941 | -0.317 |
| Head width (mm) | 0.194 | -0.292 | 0.936 |
| Lower hindlimb length (mm) | 0.974 | 0.168 | -0.149 |
| % variance explained | 94.1 | 3.5 | 2.4 |

**Table S5. Results of Tukey’s HSD test for genome mapping and coverage of the green anole (*Anolis carolinensis*).**

| **Morphological variable** | **Population 1** | **Population 2** | **Difference** | ***P* value** |
| --- | --- | --- | --- | --- |
| Mapping score | Chichijima | Hahajima | 0.605 | 0.9346 |
|  |  | Florida | -2.1987 | 0.1676 |
|  | Hahajima | Florida | 2.8037 | 0.0518 |
| Genome coverage | Chichijima | Hahajima | -1.1955 | 0.0520 |
|  |  | Florida | 0.9193 | 0.1880 |
|  | Hahajima | Florida | -2.1148 | 0.0533 |

**Table S6. Allele frequencies of candidate genes that might be subject to selection in Ogasawara Islands (Chichijima and Hahajim) and Florida populations.** 1, The average frequencies of all the SNPs within the gene. 2, The frequencies of alleles whose frequencies were major in the Ogasawara Islands, but minor in the Florida populations.

|  | Ogasawara | | Florida | |
| --- | --- | --- | --- | --- |
|  | Average frequencies^1^ | Selected allele frequencies^2^ | Average frequencies^1^ | Selected allele frequencies^2^ |
| *gadl1* | 0.6154 | 0.8264 | 0.4239 | 0.1597 |
| *ntn1* | 0.5525 | 0.7277 | 0.3317 | 0.1964 |
| *pik3cb* | 0.4176 | 0.9188 | 0.294 | 0.25 |
| *mycbp2* | 0.4901 | 0.7344 | 0.3674 | 0.0938 |
| *acot11* | 0.5391 | 1 | 0.4766 | 0.1875 |
| *foxred2* | 0.35 | 0.75 | 0.35 | 0.1875 |
| *cacng2* | 0.4161 | 0.7563 | 0.2303 | 0.15 |
| *nav3* | 0.5557 | 0.8229 | 0.4052 | 0.1354 |
| *nebl* | 0.5701 | 0.9072 | 0.4056 | 0.0625 |
| *fam188a* | 0.5729 | 0.9062 | 0.2685 | 0.25 |
| *itga8* | 0.3998 | 0.9297 | 0.2995 | 0.2969 |
| *fhod3* | 0.5819 | 0.9479 | 0.3101 | 0.3073 |
| *pamr1* | 0.2419 | 0.9063 | 0.2647 | 0.2541 |
| *unknown* | 0.2911 | 0.8958 | 0.2697 | 0.25 |
| *mical3* | 0.4496 | 0.9167 | 0.4134 | 0.1458 |

**Figure S1.** Bayesian ND2 phylogeny using Chichijima, Hahajima, and northern Florida populations in addition to haplotypes used by Campbell-Staton *et al*. (2012).


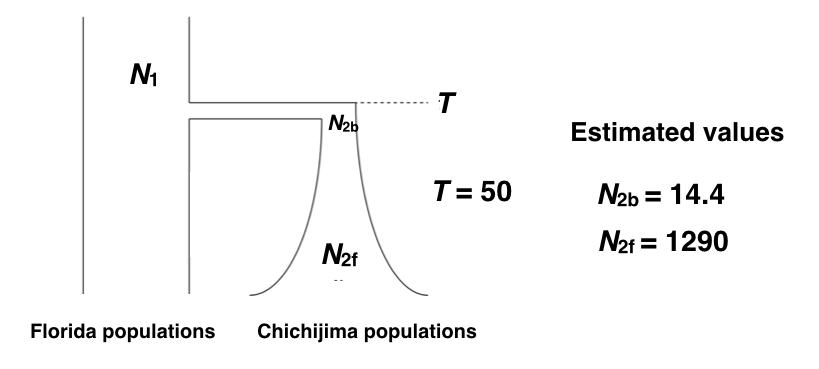


**Figure S2. Model of historical population size for Florida and Chichijima populations and the estimated values using joint site frequency distribution**. The native population size (*N_1_*) and the time of divergence between introduced and native population (generations) (*T*) was set at 35,000 and 50, respectively; *N_2b_*, the initial population size of the introduced population; *N_2f_*, the present population size of the introduced population.


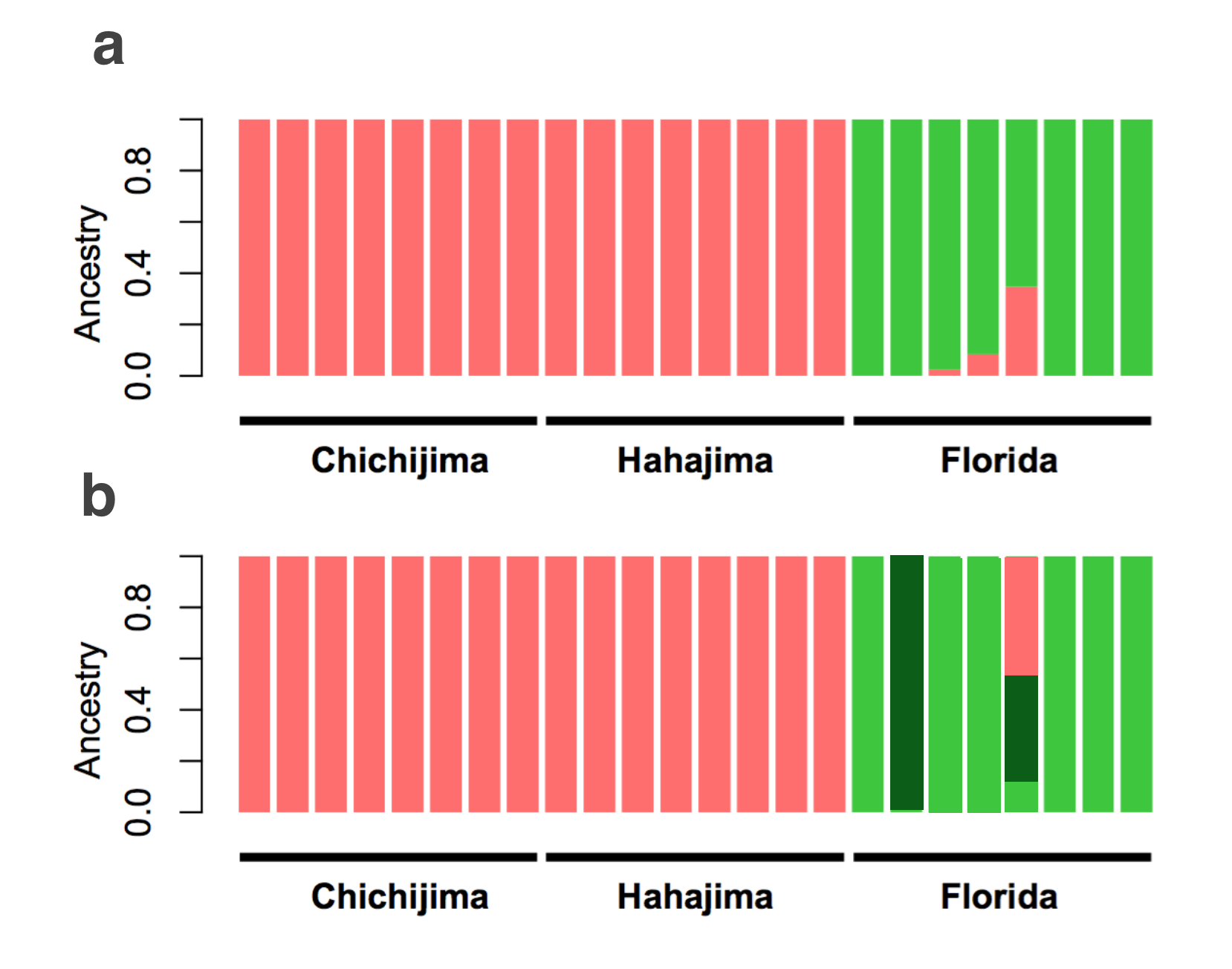


**Figure S3.** Bar plots of individual-ancestry estimates from ADMIXTURE software program using genome wide SNPs data. a) *K* = 2, b) *K* = 3. The reconstruction at *K* = 2 is the smallest cross-validation error. CV error values of *K* = 1, *K* = 2, *K* = 3, *K* = 4 and *K* = 5 were 47321, 0.46841, 0.50789, 0.58123 and 0.80049, respectively.


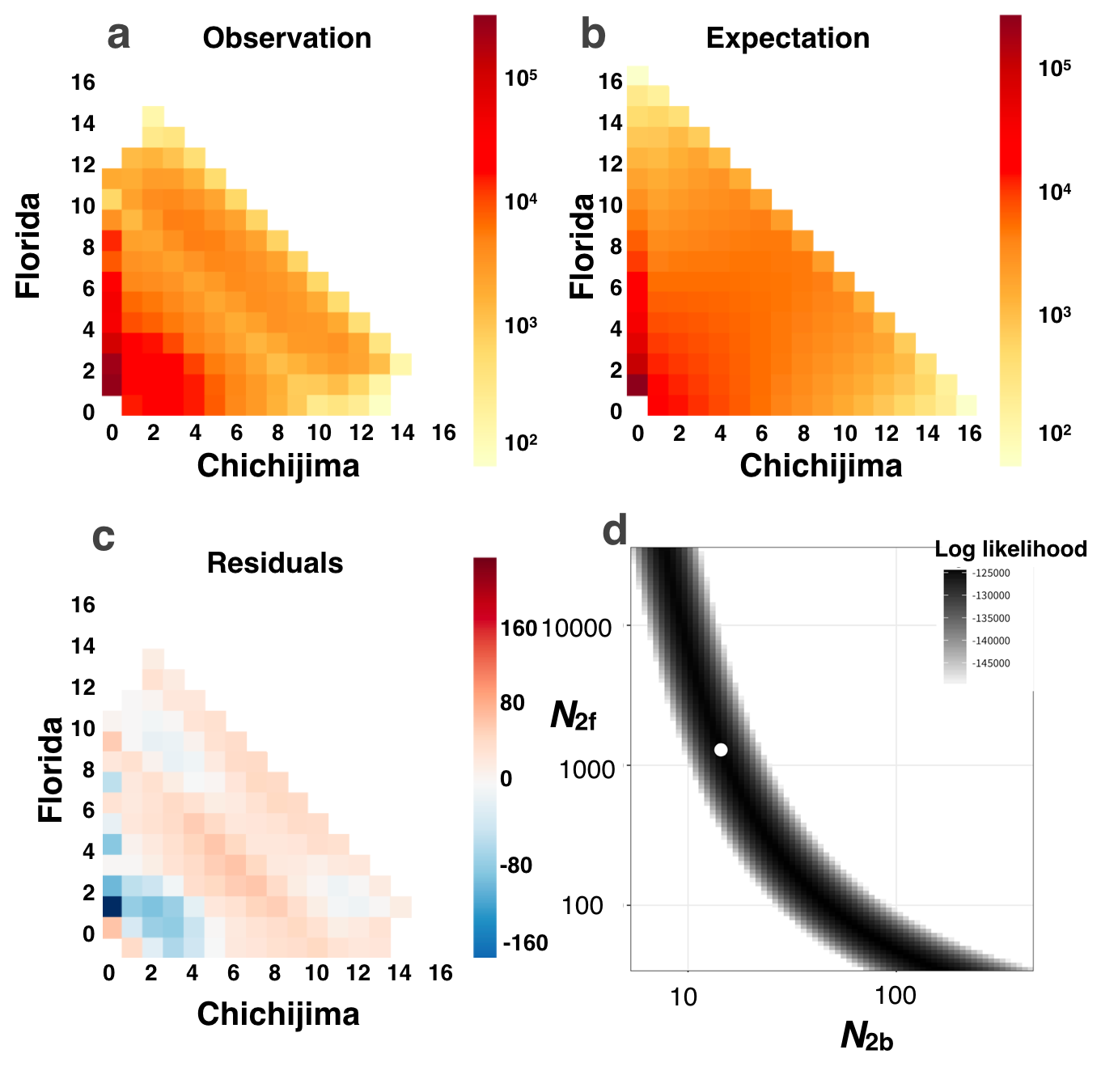


**Figure S4. Joint allele frequency distributions and log-likihood of estimated parameters of the green anole (*Anolis carolinensis*).** (a) Joint allele frequency spectrum (AFS) of 1,242,857 SNPs observed in Chichijima and Florida populations. (b) Joint AFS expected from the demographic model and the estimated parameters described in Figure 2b. (c) Residuals between a and b, which were calculated as (expected - observed) / √expected. (d) Distribution of log-likelihood within the space of demographic parameters. A white dot denotes the parameter with maximum likelihood. *N*_2b_, the initial population size of the introduced population; *N*_2f_, the present population size of the introduced population.


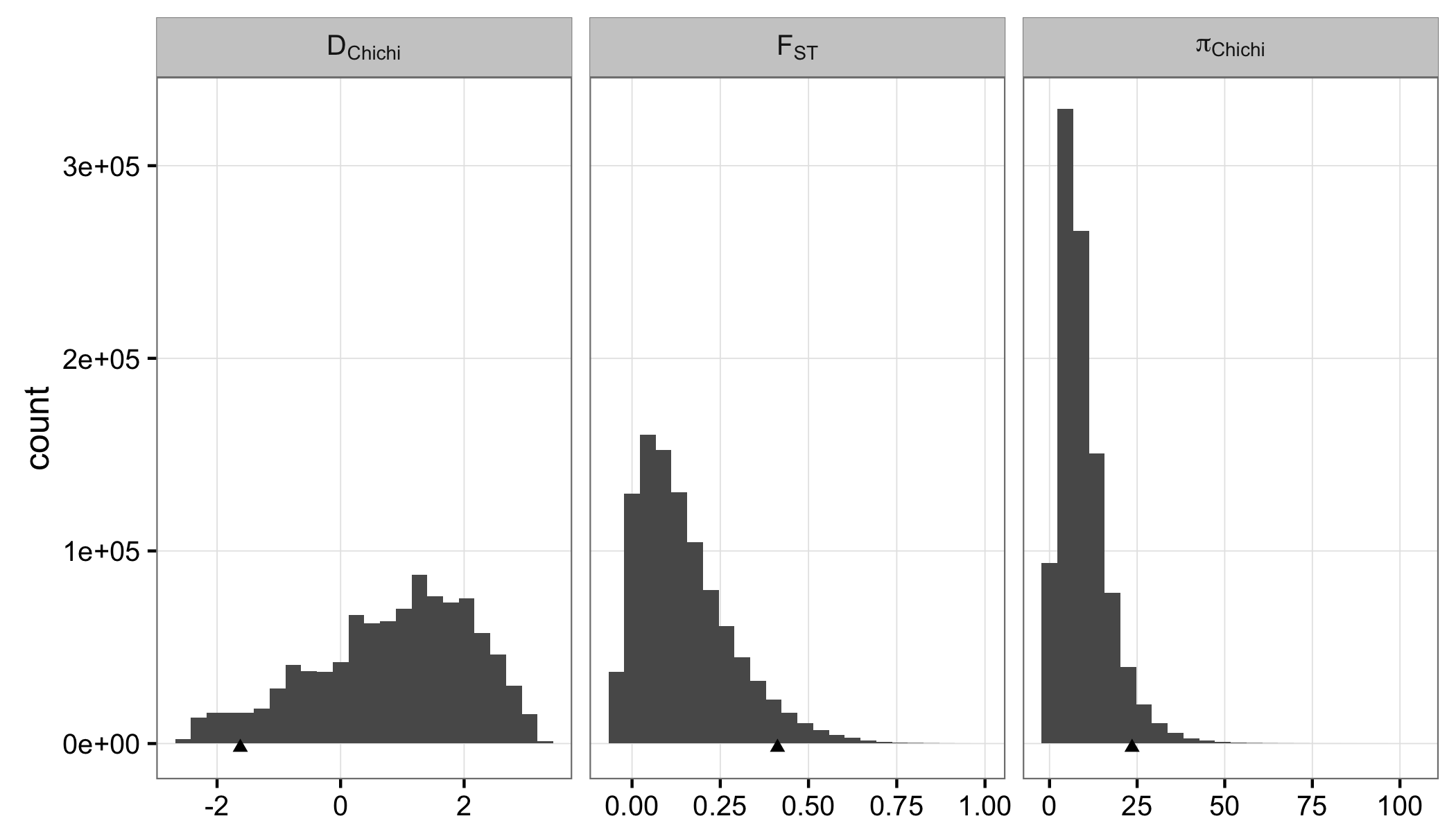

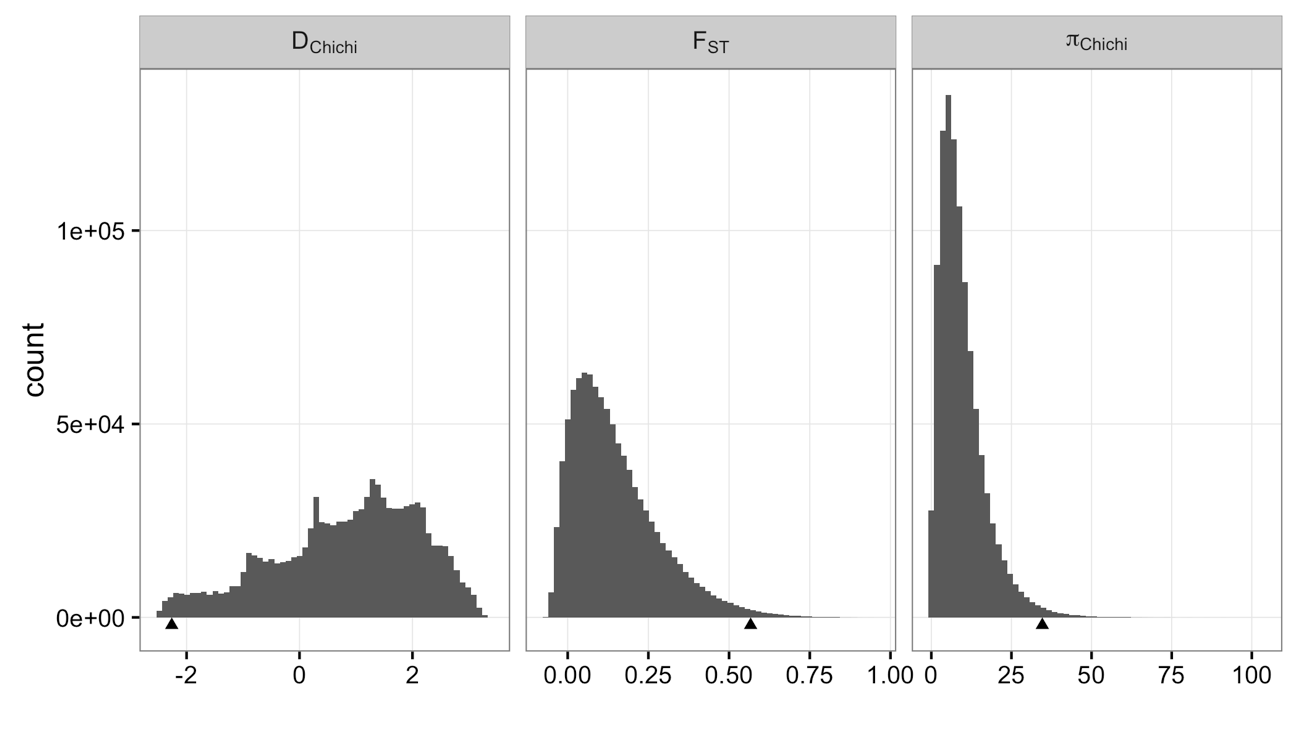


**Figure S5. The null distributions of Tajima’s D (a), *F_ST_* (b), and (c) nucleotide diversity (c) in 10kb sequences generated by coalescent simulations of the green anole (*Anolis carolinensis*)**. The coalescent simulations were conducted with the demographic parameters described in Figure 2b. Black triangles denote the 5th or 95th percentile to detect divergent selection.

**Figure S6. The distribution of Tajima's D in the 10kb sliding windows of the Florida samples of the green anole (Anolis carolinensis).**
